# Potential Applications of DNA, RNA and Protein Biomarkers in Diagnosis, Therapy and Prognosis for Colorectal Cancer: A Study from Databases to AI-Assisted Verification

**DOI:** 10.3390/cancers11020172

**Published:** 2019-02-01

**Authors:** Xueli Zhang, Xiao-Feng Sun, Bairong Shen, Hong Zhang

**Affiliations:** 1 School of Medicine, Institute of Medical Sciences, Örebro University, SE-70182 Örebro, Sweden; zhang.xueli@oru.se; 2 Centre for Systems Biology, Soochow University, Suzhou 215006, China; 3 Department of Oncology and Clinical and Experimental Medicine, Linköping University, SE-58183 Linköping, Sweden

**Keywords:** DNA, RNA, protein, single-biomarkers, multiple-biomarkers, cancer-related pathways, colorectal cancer

## Abstract

In order to find out the most valuable biomarkers and pathways for diagnosis, therapy and prognosis in colorectal cancer (CRC) we have collected the published CRC biomarkers and established a CRC biomarker database (CBD: http://sysbio.suda.edu.cn/CBD/index.html). In this study, we analysed the single and multiple DNA, RNA and protein biomarkers as well as their positions in cancer related pathways and protein-protein interaction (PPI) networks to describe their potential applications in diagnosis, therapy and prognosis. CRC biomarkers were collected from the CBD. The RNA and protein biomarkers were matched to their corresponding DNAs by the miRDB database and the PubMed Gene database, respectively. The PPI networks were used to investigate the relationships between protein biomarkers and further detect the multiple biomarkers. The Kyoto Encyclopaedia of Genes and Genomes (KEGG) pathway enrichment analysis and Gene Ontology (GO) annotation were used to analyse biological functions of the biomarkers. AI classification techniques were utilized to further verify the significances of the multiple biomarkers in diagnosis and prognosis for CRC. We showed that a large number of the DNA, RNA and protein biomarkers were associated with the diagnosis, therapy and prognosis in various degrees in the CRC biomarker networks. The CRC biomarkers were closely related to the CRC initiation and progression. Moreover, the biomarkers played critical roles in cellular proliferation, apoptosis and angiogenesis and they were involved in Ras, p53 and PI3K pathways. There were overlaps among the DNA, RNA and protein biomarkers. AI classification verifications showed that the combined multiple protein biomarkers played important roles to accurate early diagnosis and predict outcome for CRC. There were several single and multiple CRC protein biomarkers which were associated with diagnosis, therapy and prognosis in CRC. Further, AI-assisted analysis revealed that multiple biomarkers had potential applications for diagnosis and prognosis in CRC.

## 1. Introduction

Colorectal cancer (CRC) is one of the most common types of malignancies and third leading cause of cancer-related death [1]. In 2017, there were 135 430 individuals who were diagnosed for CRC and 50 260 dead from CRC only in the United States of the America [2]. Accumulating evidence has shown that the outcome of CRC is clearly dependent on the cancer stage [2,3] and follows the strict rule: early diagnosis with better survival and later diagnosis with worse prognosis [4]. If the CRC patients are diagnosed at stage I cancer the 5-year survival rate is more than 90%, while for the stage IV patients the 5-year survival is around 10% [5]. However, more than 50% of CRC patients are already in stage III + IV at diagnosis [2]. This means that they have already passed the golden diagnostic time: early diagnosis. The rule for better cancer therapy is that it is always more complicated to treat the later stages of the cancers than to treat the early cancer patients [5]. Therefore, we lose the best therapy opportunity for the CRC patients when the golden diagnosis has been missed. Although advanced cancer therapeutic techniques have improved the outcome of cancer patients, the individuals with the same types of cancer respond remarkably differently to the same therapies. A group of cancer may respond very well to the therapy, another group may not respond to the same therapy at all and even some patients will die due to the side effects of the therapy.

Studies have shown that there is great variation among patients concerning cancer therapy and patient survival [6]. During the last decades, the publications concerning genomics, proteomics and molecular pathology have reported a large amount of cancer biomarkers from a plenty of studies from various laboratories. However, there are still huge gaps between the results from the research benches to clinical bedsides. In order to understand how and when the biomarkers can be integrated into clinical practice it is crucial to translate the laboratory results into reality. More accurate early diagnosis and individual therapy will lead us to the better cancer therapy and further improve cancer patient survival [7,8].

Recently, numerous CRC-related biomarkers have been identified and hundreds of these biomarkers have been found to be associated with early diagnosis, therapy and survival of CRC [9]. The knowledge concerning applications of the biomarkers has been considered as one of the most optimal alternative way to improve the diagnosis, therapy and prognosis for CRC [10]. The development of bioinformatics, computer science and computer-assisted biomarker analysis techniques have proven very useful tools for further biomarker investigations [11]. Consequently, several biomarker databases concerning various diseases have been created which provide a large amount of valuable data to further study the functions, interactions and even applications of biomarkers in various diseases [12,13,14,15]. However, there is no such public database focusing only on CRC biomarkers and providing comprehensive information and overview of the CRC biomarkers for both basic and clinic studies. With this question in our minds, we have recently established a CRC biomarker database (CBD: http://sysbio.suda.edu.cn/CBD/index.html) [9].

In this study, we used the biomarker data from our CBD database and other public databases to analyse the aspects of the potential applications of DNA, RNA and protein biomarkers focusing in diagnosis, therapy and prognosis for CRC. AI-assisted classification techniques were used to verify the diagnostic and prognostic significances of the single and multiple biomarkers for CRC. We attempted to further clarify the important single and multiple biomarkers as well as biomarker pathways from the laboratory benches to the clinical bedside and to provide more precise criteria in diagnosis, therapy and prognosis and to benefit the CRC patients.

## 2. Results

### 2.1. Applications of CRC Biomarkers and Their Interactions in Cancer Diagnosis, Therapy and Prognosis

**Figure 1:**
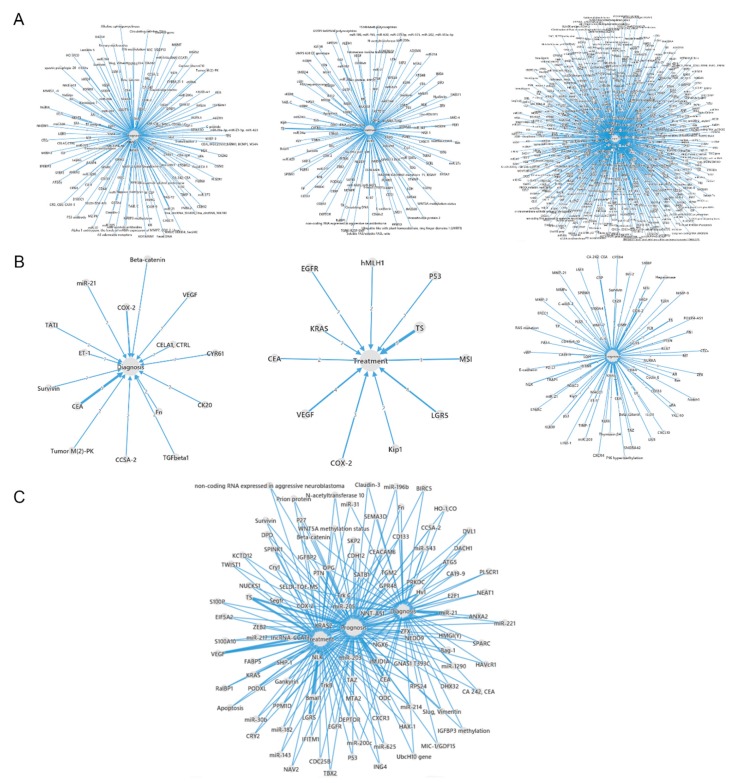
Distributions and interactions of CRC diagnosis, therapy and prognosis biomarkers from the CBD. The numbers mentioned on the lines means the amounts of articles for the correlated biomarkers. (**A**) The CRC biomarkers were classified according to their functions of diagnosis, therapy and prognosis. (**B**) The biomarkers reported by more than 2 articles are presented. (**C**) The interactions of diagnosis, therapy and prognosis biomarkers.

Applications of CRC biomarkers and their interactions in diagnosis, therapy and prognosis and relationships of the biomarkers to the diagnosis, therapy and prognosis were analysed. As shown in Figure 1A, there were 157 biomarkers which were associated with CRC diagnosis, 152 biomarkers were related to cancer therapy and 707 with cancer prognosis. According to frequency of CRC biomarkers from our database, the sub networks were reconstructed by biomarkers in the high frequency research articles. According to Figure 1B, among the 157 diagnostic biomarkers the most common biomarkers were carcinoembryonic antigen (CEA) and cyclooxygenase-2 (COX-2). For the therapy biomarkers, thymidylate synthase (TS), leucine-rich repeat-containing G protein-coupled receptor 5 (LGR5) and vascular endothelial growth factor (VEGF) were the common ones. CEA most frequently prognostic biomarkers. Interactions among the diagnostic biomarkers, therapeutic biomarkers and prognostic biomarkers were further analysed and the interactions of the multiple functional biomarkers were presented in Figure 1C.

### 2.2. Applications of PPI Networks for CRC Diagnostic, Therapeutic and Prognostic Protein Biomarkers

**Figure 2:**
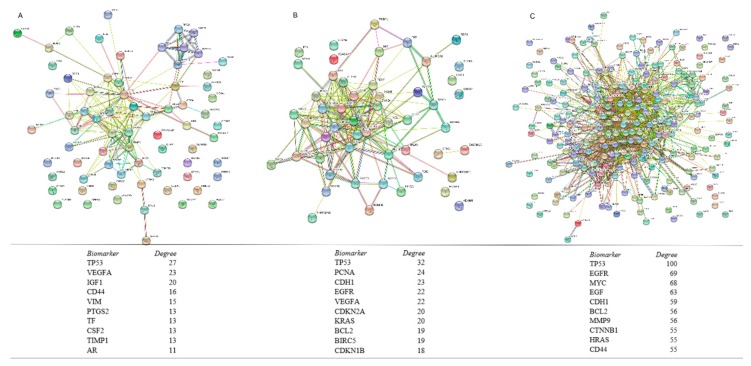
PPI networks of CRC protein biomarkers in diagnosis, therapy and prognosis. Distributions of protein biomarkers in diagnosis (**A**), therapy (**B**) and prognosis (**C**) of CRC are displayed. Top 10 most frequent protein biomarkers in related to the diagnosis, therapy and prognosis of the CRC are listed.

As shown in Figure 2, the CRC protein biomarkers were further analysed in the PPI networks for CRC diagnosis, therapy and prognosis. The biomarkers with the highest degree for the diagnosis were TP53, VEGF, IGF1 and CD44 (Figure 2A), for therapy were TP53, PCNA, CDH1 and so forth, (Figure 2B) and for prognosis were TP53, EGFR, MYC and so forth, (Figure 2C). TP53 was found as the biomarker with highest degree for all CRC diagnosis, therapy and prognosis. EGFR, Ras, CDH1 and BCL2 have been related to both CRC therapy and prognosis. (KRAS protein with therapy and HRAS protein with prognosis) CD44 is associated with both CRC diagnosis and prognosis. Most of the protein biomarkers were associated with CRC prognosis. The top 10 high degree protein biomarkers in each PPI network are selected and presented in Figure 2.

We utilized KEGG pathway enrichment to further analyse the top 10 pathways in related to diagnosis, therapy and prognosis in CRC, respectively. Results are shown in Table 1. The top enriched pathways for CRC diagnosis were Ribosome, Pathway in cancer, HIF-1 signalling pathway, Wnt signalling pathway and MicroRNAs in cancer (Table 1A). The pathways for CRC therapy were Pathways in cancer, Bladder cancer, MicroRNAs in cancer, Hepatitis B and Colorectal cancer (Table 1B). Moreover, the pathways for CRC prognosis were MicroRNAs in cancer, bladder cancer, Pathway in cancer, p53 signalling pathway and HTL V-I infection (Table 1C). Pathways in cancer and microRNAs in cancer shared essential roles in CRC diagnosis, therapy and prognosis.

The CRC biomarkers in functional pathways were further analysed by GO analysis and the results showed GO annotation in biological process for diagnosis, therapy and prognosis biomarkers (Table 2). In the CRC diagnosis, phosphorylation was an important functional pathway, such as Positive regulation of phosphorylation, Positive regulation of phosphate metabolic process, Positive regulation of protein phosphorylation and Protein complex subunit organization (Table 2A). For CRC therapy, Negative regulation of cell death, Regulation of apoptotic processes, Response to abiotic stimulus, Regulation of cell death and Negative regulation of apoptotic processes (Table 2B). Regulation of cell proliferation, Response to stress, System development, Positive regulation of cellular processes and Negative regulation of cellular processes seemed playing important roles (Table 2C). Phosphorylation was essential for CRC diagnosis. Regulation of cellular death was critical for CRC therapy. Regulations for cell proliferation and cellular processes were important for CRC prognosis. It seems that different groups of cellular functional pathways play their unique roles for CRC diagnosis, therapy and prognosis, respectively.

However, when we further estimated molecular functions of the CRC biomarkers and their pathways associated with CRC diagnosis, therapy and prognosis with GO analysis the results (Table 3) showed that protein binding, identical protein binding, binding and enzyme binding are the four pathways shared in CRC diagnosis (Table 3A), therapy (Table 3B) and diagnosis (Table 3C). Cellular Component GO analysis for the CRC biomarkers and pathways revealed in Table 4 that CRC diagnosis and prognosis biomarkers shared extracellular space, vesicle, extracellular region and extracellular region part pathways.

### 2.3. CRC Biomarkers in Pathway in Cancer and miRNAs in Cancer Pathway

**Figure 3:**
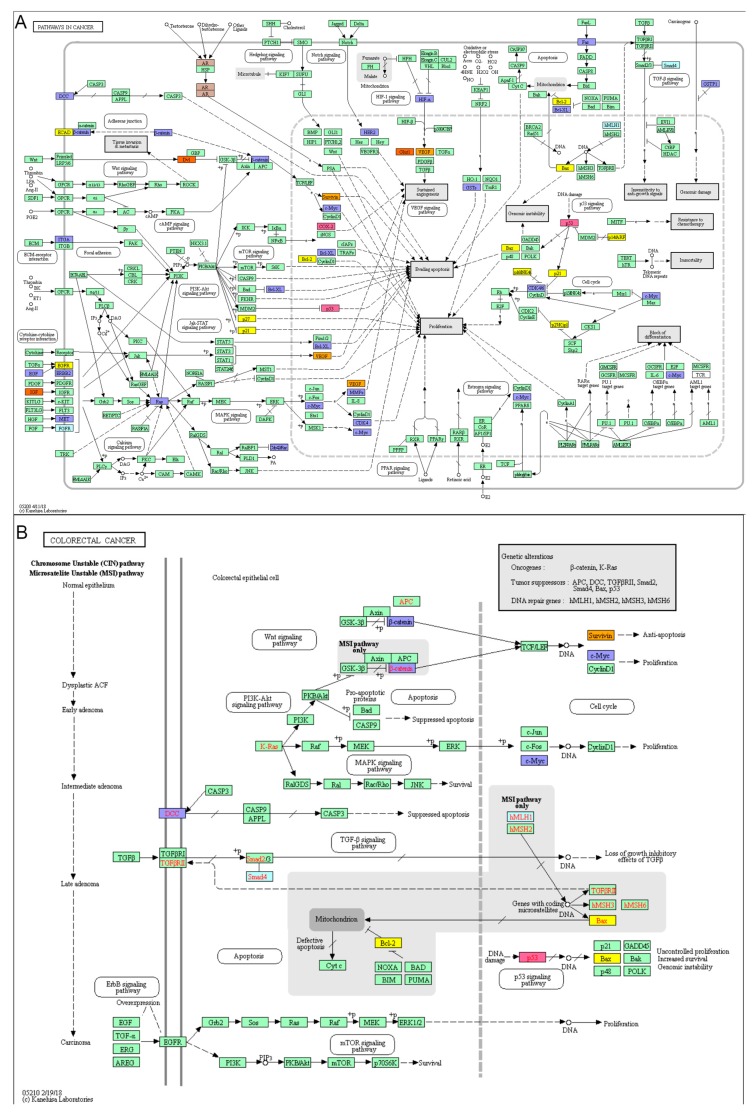
Biomarkers in the Pathways in cancer. (**A**) Various cancer pathways involve in different cancer initiation and progression. (**B**) CRC biomarkers for diagnosis, therapy and prognosis biomarkers in the CBD were mapped in different colours in Pathways in cancer. The CRC biomarkers have been associated with apoptosis, cell proliferation, VEGF signalling pathway and Ras signalling pathway in the Pathways in cancer. Red: diagnosis biomarker; Blue: treatment biomarker; Purple: prognosis biomarker; Orange: diagnosis & treatment biomarker; Yellow: treatment & prognosis biomarker; Pink: diagnosis & treatment & prognosis biomarker.

CRC biomarkers were analysed in association with Pathways in cancer (Figure 3). There were many biomarkers and pathways which are found in the Pathways in cancer which were associated with CRC. However, the most common and important pathways were p53, Ras and PI3K and apoptosis, cell proliferation and angiogenesis pathways.

**Figure 4:**
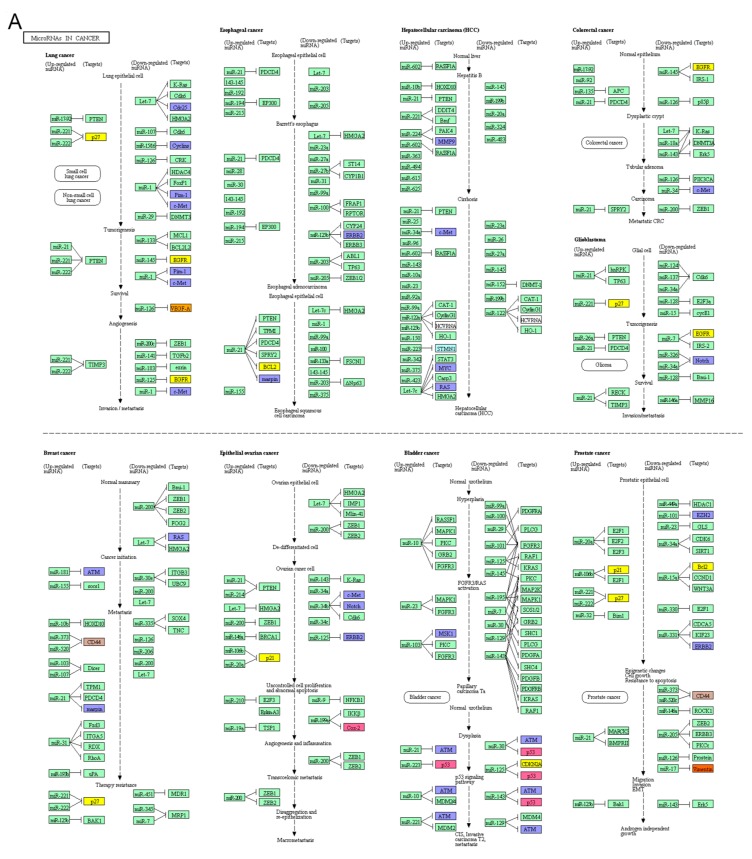

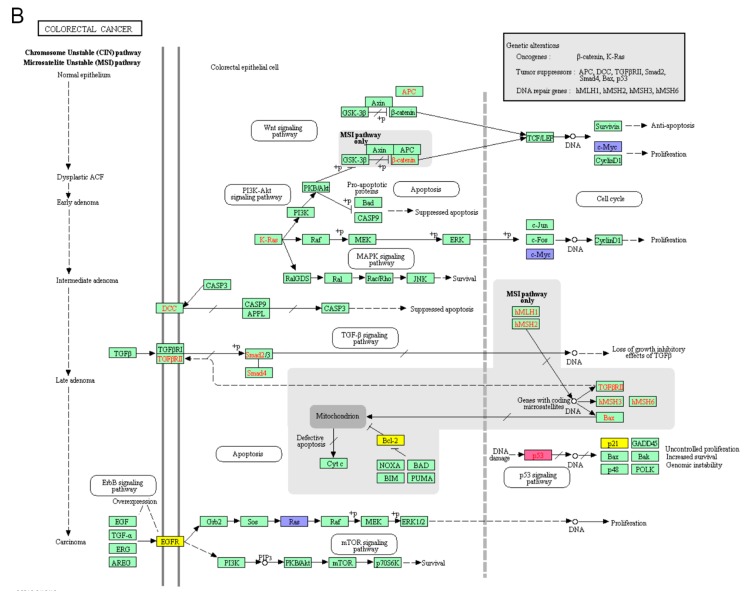
MiRNA in cancers. (**A**) MiRNAs involve in different types of cancers. (**B**) CRC Biomarkers in the miRNAs in cancer pathway. Different miRNAs and interactions among the miRNAs and a variety of genes, such as APC and K-ras have been involved in CRC initiation and progression process.

CRC miRNA biomarkers in the miRNAs in cancer pathway have been closely associated with the Vogelstein’s CRC developing model. Different miRNAs and interactions among the miRNAs and a variety of genes, such as APC and K-ras have been involved in CRC initiation and progression process. MiR-135 inhibits APC at CRC initiating level; Let-7, miR-18a and miR-143 inhibit K-ras at CRC progression level; miR-21 and miR-200 involve in the CRC metastasis (Figure 4).

### 2.4. miRNAs and Proteins Biomarkers for CRC Diagnosis, Therapy and Prognosis

**Figure 5:**
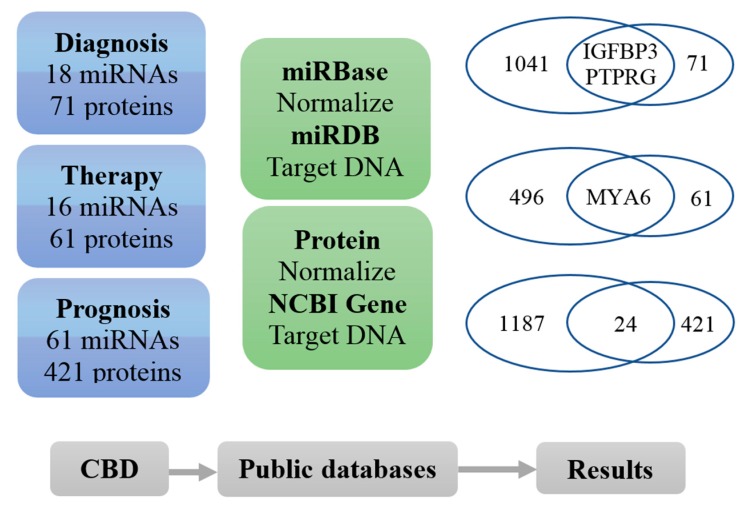
Associations of DNA, RNA and protein biomarkers in diagnosis, therapy and prognosis of CRC. The RNA and protein biomarkers from our CBD were converted to their corresponding genes and the relationships between the overlapping genes were further analysed. There were two genes (IGFBP1 and PTPRG) from both RNA and protein biomarkers which were associated to CRC diagnosis and one gene (MYA6) was related to therapy. However, there were 24 genes which were associated with prognosis.

As shown in Figure 5, we analysed miRNA and protein biomarkers concerning CRC diagnosis, therapy and prognosis in our CBD database and found that there are 16 miRNA and 71 protein biomarkers for diagnosis in the CBD database. After standardization through miRBase (http://www.mirbase.org/) and NCBI protein database (https://www.ncbi.nlm.nih.gov/protein), the miRNAs and proteins were converted to their corresponding target DNAs in the miRDB database and NCBI Gene database. 1041 target genes in the miRDB were found for their 18 diagnosis miRNA biomarkers in our CBD and 71 corresponding genes in the NCBI Gene database were found for the 71 diagnostic protein biomarkers in the CBD. The converted DNAs for diagnostic miRNA and protein biomarkers were overlapped in the check points IGFBP3 and PTPRG. For the CRC therapy biomarkers, there were 16 miRNAs and 61 proteins. After the standardization and converting to DNAs, MYA6 was found as the check point for both miRNAs and proteins for CRC therapy. There were 61 miRNAs and 421 proteins were found as the CRC prognostic biomarkers in our CBD database. After the standardization and converting to their corresponding DNAs, 24 check points were found to associate with CRC prognosis between 1187 for miRNAs and 421 for proteins.

### 2.5. Prognostic DNA Biomarkers in CRC

For prognosis biomarkers, the protein-miRNA biomarkers overlapping genes are as follows: ATP11A, CASK, CD44, DEK, DUSP5, DYRK2, EIF5A2, EPAS1, HOXB7, KRAS, MACC1, NRCAM, PRRX1, PTEN, RALBP1, S1PR1, SATB1, SLIT2, STAT3, TAGLN2, TBL1XR1, ZEB1, ZEB2, ZFX. After searching in the CBD we find that KRAS gene has been reported as DNA biomarker in CRC [16]. The biological analysis results for these overlapped DNA are shown in Table 5.

**Figure 6:**
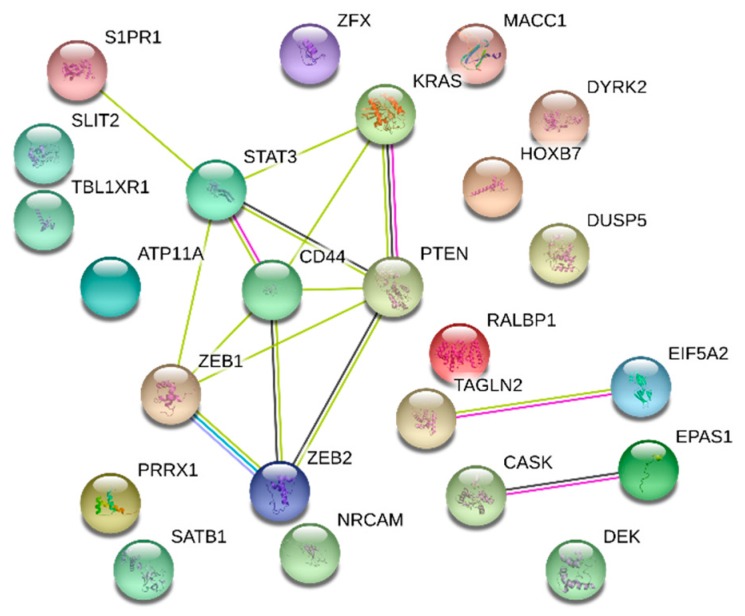
PPI network for the 24 overlapping prognosis genes. There were 13 genes which can been used to predict patients survival. The remaining genes worked in pairs or in groups to predict the prognosis.

In order to find the relationship of the CRC prognostic biomarkers and the prognostic DNA biomarkers in CRC were mapped in PPI network (Figure 6). There were many single genes which were confirmed to be associated in the PPI networks. We showed also 15 significant gene interactions such as KRAS/PTEN and ZEB1/ZEB2 in the PPI networks, which may serve as combined biomarkers.

### 2.6. Verifications of Protein Biomarkers in Diagnosis and Prognosis

**Figure 7:**
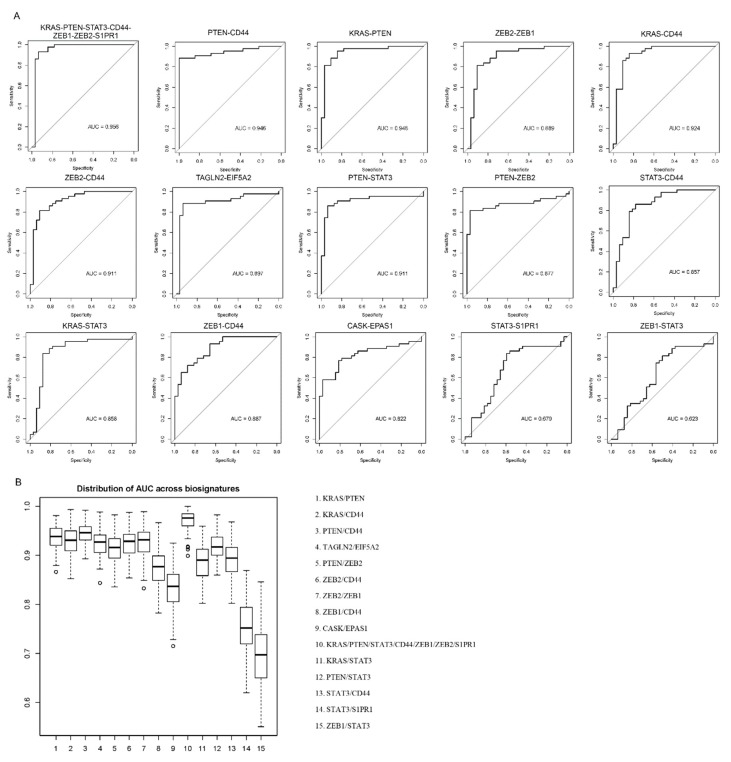
Diagnostic performance of multiple biomarkers for CRC. (**A**) The receiver operating (ROC) curves of all the 15 multiple biomarkers. (**B**) Distributions of AUC across biosignatures. The area under curve (AUC) statistics from 100 random training/testing divisions. The 15 multiple biomarkers were ranked.

AI-assisted classification techniques were utilized to further verify the significance of the 15 commonly combined multiple biomarkers predicted from PPI networks in diagnosis and prognosis for CRC. In Figure 2 we showed that many biomarkers can be applied in more than one ways along diagnosis, treatment and prognosis. So the diagnostic value for these 15 multiple biomarkers were further analysed. Figure 7 revealed the diagnostic ROC curves and distributions of AUC across biosignatures of the combined multiple protein biomarkers in CRC. The combined multiple protein biomarker of KRAS-PTEN-STAT3-CD44-ZEB1-ZEB2-S1PR1 had the most significant value amount the 15 combined biomarkers and it played the most significant role in CRC diagnosis.

**Figure 8:**
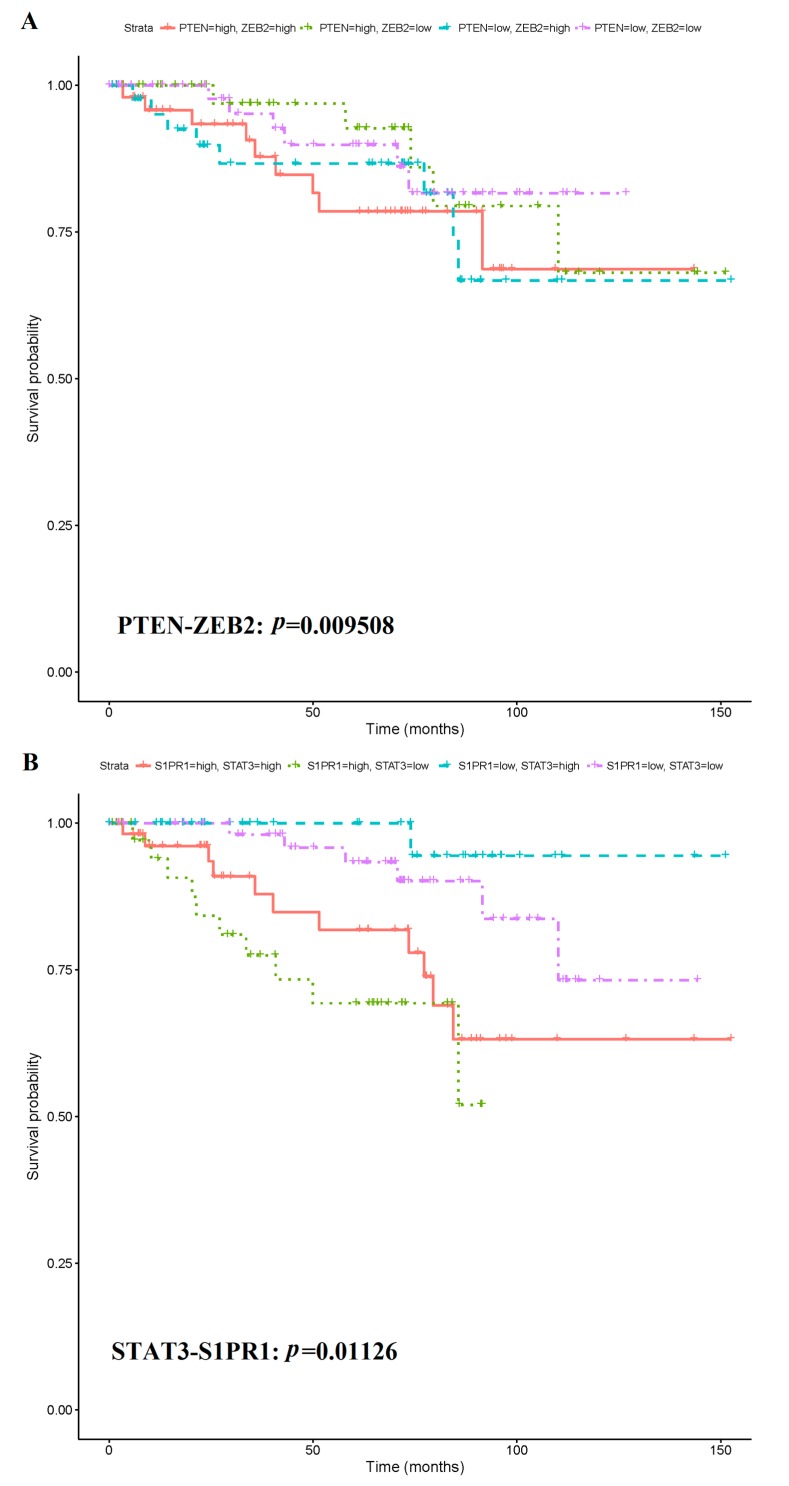

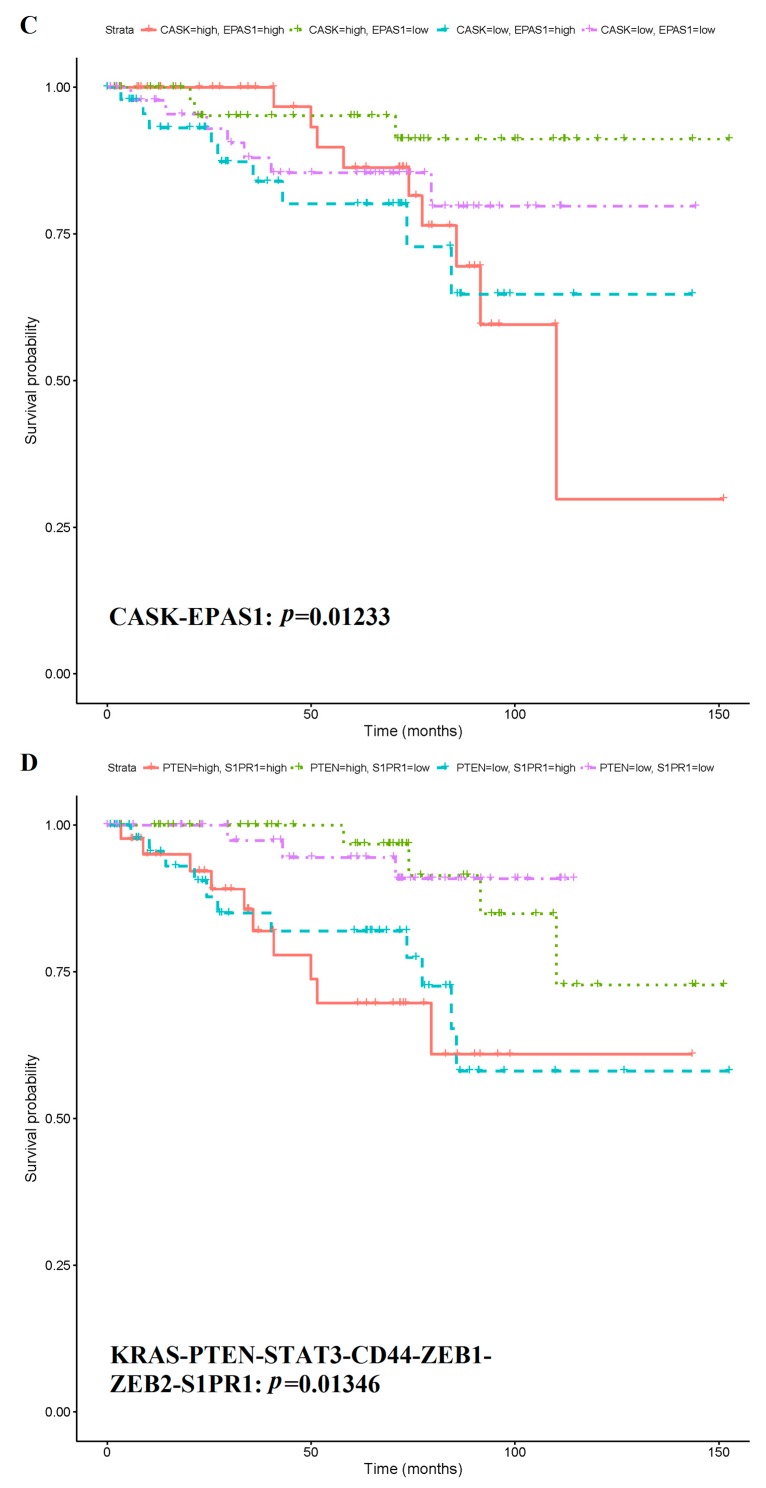

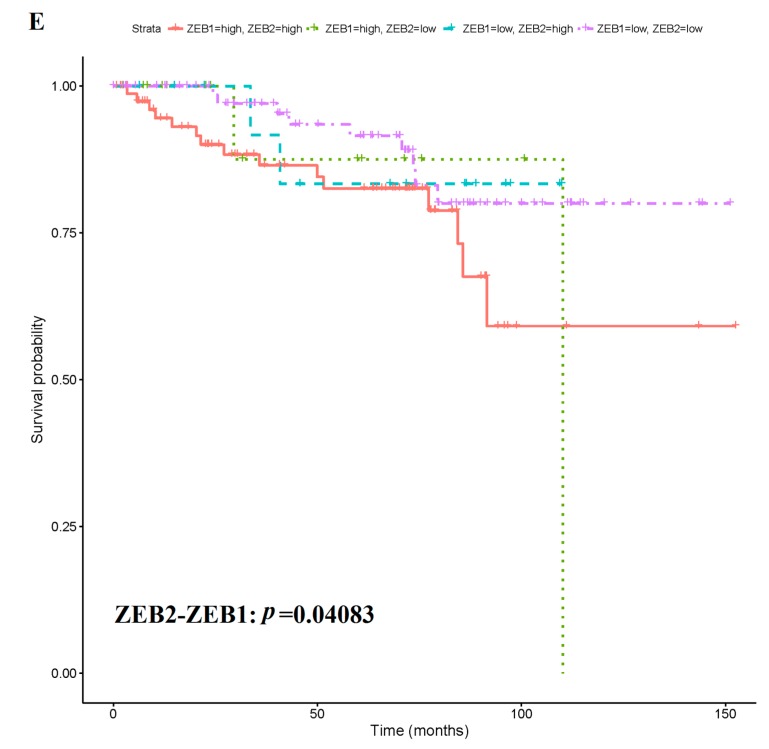
Kaplan-Meier survival curves of five multiple biomarkers with significant prognosis value. (**A**) Kaplan-Meier survival curves of multiple biomarker combined by PTEN and ZEB2. (**B**) Kaplan-Meier survival curves of multiple biomarker combined by STAT3 and S1PR1. (**C**) Kaplan-Meier survival curves of multiple biomarker combined by CASK and EPAS1. (**D**) Kaplan-Meier survival curves of multiple biomarker combined by KRAS, PTEN, STAT3, CD44, ZEB1, ZEB2 and S1PR1. (**E**) Kaplan-Meier survival curves of multiple biomarker combined by ZEB2 and ZEB1.

AI-assisted prognosis analysis showed that five of the 15 combined had statistical significance to predict CRC prognosis. Of these, 5 biosignatures were significant at a level of 0.05 using the log-rank test. After multiplicity correction using the Holm FWER correction, a single biosignature was significant, the PTEN-ZEB2 pair. Its corresponding Log rank Score is 9.31. Further analyses revealed that the CRC patients with lower S1PR1 levels had better prognosis and those with higher S1PR1 levels had worse prognosis, independent of PTEN and STAT3 (Figure 8).

## 3. Discussion

In the CBD database [9] we have collected all the reported CRC biomarkers from the PubMed, which has provided a useful platform for CRC researchers to further investigate the effects of the biomarkers in early diagnosis, beneficial therapy and improved prediction for CRC patient survival. In this study, the potential applications of CRC biomarkers and their interactions in cancer diagnosis, therapy and prognosis and relationships of the biomarkers among the diagnosis and prognosis were further analysed and verified by AI-assisted techniques. We found there were several single and multiple functional biomarkers which are important in diagnosis, therapy and prognosis for CRC.

Although accumulating evidence concerning studies of biomarkers in cancers have been focused on cancer diagnosis, therapy and prognosis there are only few biomarkers which have been clinically utilized for early diagnosis, selecting the suitable cancer patients for better therapy and predicting prognosis. In this study, the applications of the CRC biomarkers in diagnosis, therapy and prognosis were investigated at cellular, molecular and pathway levels to further understand the biological and molecular process of the biomarkers. GO analysis showed that various biological processes, such as molecular functions and cellular composition of the protein biomarkers are involved in CRC diagnosis, therapy and prognosis. Protein phosphorylation and cell proliferation have been associated with the CRC diagnosis. Cell death and apoptosis are related to the CRC therapy and cell proliferation and biological process to the CRC prognosis. We provided clear evidence from molecular pathways and cell biology levels that the CRC biomarkers can be utilized to early diagnosis, better therapy and predicting patients outcome.

CRC biomarkers in various molecule networks and biological pathways are important for CRC. In this study, we showed the top enriched pathways in diagnosis, therapy and prognosis with the KEGG enrichment analysis. The Pathways in cancer and miRNA in cancer pathway are the most common pathways for the CRC biomarkers. As expected, the CRC biomarkers have been mainly working for the molecular binding and there are the similar pathways for the molecular binding function of CRC protein biomarkers. In the biological processes, most of annotated pathways are positive regulators for diagnosis and prognosis biomarkers and negative regulators for therapy biomarkers, indicating that protein biomarkers play different roles in CRC diagnosis, therapy and prognosis.

Proteins are the major consistency of CRC biomarkers and biological functions are always implemented by several different proteins. In this study, we collected all the protein biomarkers from our CBD [9] and drew PPI networks concerning diagnosis, therapy and prognosis, respectively. Most of the protein biomarkers were connected to the PPI networks. There were several protein biomarkers which acted as essential hubs in all the three PPI networks, such as TP53, EGFR, CDH11 and BCL2. GO analysis showed that these proteins played an important role in positive regulation of intracellular transportation, cellular protein localization and cell-cell adhesion, which provided the evidence that our future study should focus on such hub proteins as the biomarkers for CRC.

Potential applications of the CRC protein biomarkers in PPI networks for diagnostic, therapeutic and prognostic biomarkers were further analysed and we found that the most frequent protein biomarkers were associated with CRC prognosis. However, the roles of CRC protein biomarkers for diagnosis, therapy and prognosis can be overlapped with multiple functions, such as TP53 in CRC therapy and prognosis [17,18,19], Ras [20], BCL2 [21], CD44 [22], CEA [23] in CRC prognosis. The similar results from gene expression and PPI data analysis for accurate prediction have been found in leukaemia [24]. The molecular functions in protein networks of the protein biomarkers decided whether the protein biomarkers play a single or multiple roles in CRC. High degree protein biomarkers from our CRC database [9] were found to associate with p53, Ras, PI3K, apoptosis, proliferation and angiogenesis, which are the essential pathways in CRC formation, diagnosis, therapy and prognosis. We further analysed the CRC protein biomarkers from our database by KEGG pathway enrichment concerning diagnosis, therapy and prognosis, respectively. The diagnosis, therapy and prognosis protein biomarkers have been found to share the same pathways, such as pathway in cancer and microRNAs in cancer. Moreover, the CRC diagnosis protein biomarkers were enriched in the Wnt signalling pathway. The therapy-associated protein biomarkers were found in the colorectal cancer pathway and prognosis protein biomarkers in p53 signalling pathway, indicating that there are single and multiple cancer pathways which may play various role in CRC diagnosis, therapy and prognosis.

Various miRNAs and their interactions with different genes, such as APC and KRAS, have been involved in CRC initiation, development and progression processes. The miRNAs have been considered as important players in the tumorigenesis. A number of miRNAs have been identified with miRNA microarrays as potential biomarkers for cancers [25,26,27]. Different miRNAs and genes are involved in various CRC progression, such as miR-135 with APC and miR-21 with PDCD4 in the CRC initiation (Figure 4). In addition, miRNAs in cancer pathway has been related to cancer initiation, development and progression of several cancer types (Figure 4). In this study, we showed that different miRNAs played different roles in the CRC development and progression by suing NCBI, miRBase, miRDB, KEGG, GO Consortium and STRING databases which contain a huge amount of genomics and proteomics data. Systematic and integrated analyses of the CRC biomarkers in the miRNAs in cancer pathway provided an evidence the multiple miRNA biomarkers should play more critical roles in diagnosis, therapy and prognosis of CRC. Under CRC progression from the normal epithelial cells to primary and metastatic cancer cells, there are up-regulated and down-regulated miRNAs which are involved in this molecular process, such as the up-regulated miRNA-135 inhibiting expression of APC gene to block the process from the normal cells to dysplastic cells. EGFR as a therapy and prognosis biomarker and c-Met as a prognosis biomarker have both down-regulated under the CRC progression. EGFR is regulated by miR-145, which has been reported as a biomarker for acute pulmonary embolism [28], bipolar mania [29], temporal lobe epilepsy [30], breast cancer [31] and lung cancer [32]. C-Met is regulated by miR-34, which is a known biomarker in CRC, [33] indicating that different miRNAs may involve in a variety of cancer types and cancer progression in various cancer types may be regulated by the same miRNAs.

There were many protein biomarkers which were regulated by various miRNAs that identified as biomarkers for CRC. Moreover, further analyses of the relationship between protein and miRNA biomarkers showed that DNA was considered as the connection between protein miRNA biomarkers. Multiple biomarkers played better roles in the diagnosis [34,35,36], therapy [37,38] and prognosis [39,40,41] for CRC although there was disagreement concerning combination of two biomarkers [35].

In this study, we utilized AI-assisted classification techniques to further verify the significance of both the single and multiple protein biomarkers in diagnosis and prognosis for CRC. The multiple biomarkers revealed strongly statistical significance to precise diagnosis and predict prognosis in CRC and a more optimal and precise tool to investigate cancer biomarkers.

## 4. Materials and Methods

### 4.1. Data Collection and Construction of the CRC Biomarker Application Networks

870 CRC biomarkers were collected from the published articles indexed in PubMed to construct a CBD database [8]. In this study, we selected the CRC biomarkers concerning diagnosis, therapy and prognosis to produce the CRC biomarker application networks and further analyse significant importance of the biomarkers from our CBD in the diagnosis, therapy and prognosis biomarkers for CRC. The gene expression data collected from Gene Expression Omnibus (GEO) database: Series GSE87211, Platform GPL13497 were used to test the prognosis and diagnosis value of multiple biomarkers, which contains 203 rectal tumour samples and 160 control samples and was obtained from Affymetrix Human Genome arrays [42].

### 4.2. Systematic Analysis for the CRC Protein Biomarkers

In order to perform a systematic analysis for protein biomarkers, all the 583 CRC protein biomarkers from the CBD were collected to construct the protein-protein interaction (PPI) networks using the STRING database (https://string-db.org/). The relationship between the biomarkers and diagnosis, therapy and prognosis were further investigated. The pathway enrichment analysis was conducted with the Kyoto Encyclopaedia of Genes and Genomes (KEGG) database (http://www.genome.jp/kegg/) to further cluster these protein biomarkers at pathway levels. The Gene Ontology Consortium database (GO: http://www.geneontology.org/) was used to annotate the CRC protein biomarkers into corresponding pathways at three levels: biological process, cellular component and molecular function. The enriched pathways were ranked according to the false discovery rate (FDR) and gene counts.

### 4.3. Overlapping Analysis of miRNA and Protein Biomarkers

In order to make comprehensive overlapping analysis of the CRC biomarker, both miRNA and protein biomarkers were matched to their corresponding genes. The miRDB database (http://www.mirdb.org/) was utilized to assign the miRNA biomarkers to their gene targets (the genes with more than 95 target prediction score were selected). The algorithm for the prediction score (*S*) of each gene is as following:

*S* = 100 × (1 − ∏*ni* = 1*Pi*)

where n represents the number of predicted target gene sites number and Pi is statistical significance of gene sites calculated by support vector machines (SVMs) [43]. For each target gene, higher predicted score represents greater statistical confidence.

The NCBI Gene database (https://www.ncbi.nlm.nih.gov/gene) was used to match the protein biomarkers to their coding genes. The biological functions of the overlap between the genes matching the miRNA and protein biomarkers were further investigated. The STRING PPI network was utilized to analyse the relationships among the overlapping genes and to search for multiple biomarkers. The biological functions of the biomarkers were studied with KEGG pathway enrichment analysis and GO annotation.

### 4.4. AI-assisted Verification

Tissue samples were classified as cancerous according to a binary classification model. The tissue classes were normal mucosa (0) and tumour (1) tissues. The tissue class Y was modelled according to logistic regression,

$$\log\left(\frac{E(Y)}{1\;-\;E(Y)}\right)\;=\;\beta_{0}+\overset{J}{\underset{j\;=\;1}{\sum }}\beta_{j}x_{j}$$

where *p* = *E*(*Y*) is the expected proportion belonging to the tumour class and parameter *β_j_* corresponds to biomarker j.

Altogether, 15 models (multiple biomarkers found in PPI network) were considered, one for each of the candidate biosignatures. For each candidate, we randomly divided the data set into a training and testing set according to an 80/20 division. We then fit the model to the training set and evaluated the predictive performance on the testing set according to the area under the curve (AUC), a measure of a model’s ability to discriminate between classes. To evaluate the stability of each model, we replicated the above procedure 100 times to generate 100 AUC statistics for each model.

Associated with these samples were censored survival times, with the event death due to tumour being recorded. We modelled time of death due to tumour according to a Cox Proportional Hazards Model using the list of 15 biosignatures. The corresponding Kaplan-Meier survival curve test were used to estimate the statistical significances of the multiple biomarkers in CRC prognosis. When the *p*-values < 0.05, the results were considered as statistically significant.

The statistical package R (3.4.3) was used to analyses gene expression data. R-package GEOquery (2.46.15) was used to access data from the GEO repository. R-package pROC (1.10.0) was used to calculate AUCs. R-package survival was used to fit proportional hazards models. R-packages ggplot2 (2.2.1) and survminer (0.4.2) were used to produce Kaplan-Meier curves.

## 5. Conclusions

In this study, we showed the potential applications of the CRC biomarkers in diagnosis, therapy and prognosis for CRC. We reported that there were many single biomarkers which were associated with the early diagnosis, better therapy and predict prognosis in CRC. However, the combinations of multiple biomarkers and pathways might play more critical roles in diagnosis, therapy and prognosis for CRC than the single biomarkers. Therefore, the applications of multiple biomarkers and pathways could provide more precise criteria as valuable tools for early diagnosis, benefiting therapy and predicting prognosis for CRC patients.

## Figures and Tables

**Table 1:** KEGG pathway enrichment results for CRC protein biomarkers.

Pathway ID	Pathway Description	Counts	FDR
**A. KEGG pathway enrichment for diagnosis biomarkers**			
03010	Ribosome	6	0.00157
05200	Pathways in cancer	8	0.00213
04066	HIF-1 signalling pathway	5	0.00281
04310	Wnt signalling pathway	5	0.00765
05206	MicroRNAs in cancer	5	0.00803
05131	Shigellosis	3	0.049
**B. KEGG pathway enrichment for treatment biomarkers**			
05200	Pathways in cancer	15	4.52 × 10^−13^
05219	Bladder cancer	7	6.28 × 10^−10^
05206	MicroRNAs in cancer	9	8.43 × 10^−9^
05161	Hepatitis B	8	1.56 × 10^−7^
05210	Colorectal cancer	6	3.78 × 10^−7^
04110	Cell cycle	7	9.35 × 10^−7^
05218	Melanoma	6	9.35 × 10^−7^
05215	Prostate cancer	6	2.7 × 10^−6^
05212	Pancreatic cancer	5	1.48 × 10^−5^
05220	Chronic myeloid leukaemia	5	2.48 × 10^−5^
**C. KEGG pathway enrichment for prognosis biomarkers**			
05206	MicroRNAs in cancer	23	1.16 × 10^−17^
05219	Bladder cancer	13	1.47 × 10^−14^
05200	Pathways in cancer	26	3.98 × 10^−13^
04115	p53 signalling pathway	12	7.01 × 10^−10^
05166	HTLV-I infection	18	3.39 × 10^−8^
04060	Cytokine-cytokine receptor interaction	18	5.3 × 10^−8^
04151	PI3K-Akt signalling pathway	20	7.36 × 10^−8^
05215	Prostate cancer	11	1.15 × 10^−7^
05205	Proteoglycans in cancer	16	1.28 × 10^−7^

**Table 2:** GO analysis results in biological process level for CRC protein biomarkers.

Pathway ID	Pathway Description	Counts	FDR
**A. GO analysis in biological process level for diagnosis biomarkers**			
Go:0042327	Positive regulation of phosphorylation	20	6.22 × 10^−9^
Go:0045937	Positive regulation of phosphate metabolic process	21	6.22 × 10^−9^
Go:0001934	Positive regulation of protein phosphorylation	19	1.42 × 10^−8^
Go:0071822	Protein complex subunit organization	24	1.42 × 10^−8^
Go:0042127	Regulation of cell proliferation	24	2.08 × 10^−8^
Go:0042981	Regulation of apoptotic process	23	3.65 × 10^−8^
Go:0048583	Regulation of response to stimulus	34	4.31 × 10^−8^
Go:0043933	Macromolecular complex subunit organization	27	9.8 × 10^−8^
Go:0043066	Negative regulation of apoptotic process	18	1.39 × 10^−7^
Go:0008284	Positive regulation of cell proliferation	17	4.33 × 10^−7^
**B. GO analysis in biological process level for treatment biomarkers**			
GO:0060548	Negative regulation of cell death	20	7.29 × 10^−11^
GO:0042981	Regulation of apoptotic process	21	5.27 × 10^−9^
GO:0009628	Response to abiotic stimulus	19	8.77 × 10^−9^
GO:0010941	Regulation of cell death	21	8.77 × 10^−9^
GO:0043066	Negative regulation of apoptotic process	17	8.77 × 10^−9^
GO:0031325	Positive regulation of cellular metabolic process	26	8.79 × 10^−8^
GO:0010604	Positive regulation of macromolecule metabolic process	25	1.34 × 10^−7^
GO:0009893	Positive regulation of metabolic process	28	1.89 × 10^−7^
GO:0009605	Response to external stimulus	21	3.8 × 10^−7^
GO:0048523	Negative regulation of cellular process	29	4.12 × 10^−7^
**C. GO analysis in biological process level for prognosis biomarkers**			
GO:0042127	Regulation of cell proliferation	76	3.63 × 10^−29^
GO:0006950	Response to stress	100	4.56 × 10^−21^
GO:0048731	System development	101	1.33 × 10^−20^
GO:0048522	Positive regulation of cellular process	111	5.31 × 10^−20^
GO:0048523	Negative regulation of cellular process	105	5.31 × 10^−20^
GO:0031325	Positive regulation of cellular metabolic process	88	6.82 × 10^−20^
GO:0048518	Positive regulation of biological process	119	8.49 × 10^−20^
GO:0010604	Positive regulation of macromolecule metabolic process	84	2.55 × 10^−19^
GO:0048519	Negative regulation of biological process	107	7.7 × 10^−19^
GO:0051247	Positive regulation of protein metabolic process	60	1.19 × 10^−18^

**Table 3:** GO analysis results in molecular function level for CRC protein biomarkers.

Pathway ID	Pathway Description	Counts	FDR
**A. GO Analysis in molecular function level for diagnosis biomarkers**			
GO:0005515	Protein binding	44	2.81 × 10^−10^
GO:0005102	Receptor binding	20	4.2 × 10^−7^
GO:0042802	Identical protein binding	15	0.000526
GO:0005488	Binding	53	0.00127
GO:0001968	Fibronectin binding	3	0.0307
GO:0005539	Glycosaminoglycan binding	6	0.0353
GO:0003735	Structural constituent of ribosome	5	0.0358
GO:0005126	Cytokine receptor binding	6	0.0358
GO:0032403	Protein complex binding	9	0.0358
GO:0019899	Enzyme binding	14	0.0365
**B. GO Analysis in molecular function level for treatment biomarkers**			
GO:0005515	Protein binding	36	3.52 × 10^−10^
GO:0042802	Identical protein binding	16	8.85 × 10^−7^
GO:0046983	Protein dimerization activity	13	1.15 × 10^−5^
GO:0005488	Binding	42	0.000317
GO:0019899	Enzyme binding	15	0.000317
GO:0042803	Protein homodimerization activity	10	0.000445
GO:0043566	Structure-specific DNA binding	7	0.00061
GO:0046982	Protein heterodimerization activity	7	0.00061
GO:0030983	Mismatched DNA binding	3	0.000839
GO:0004861	Cyclin-dependent protein serine/threonine kinase inhibitor activity	3	0.00138
**C. GO Analysis in molecular function level for prognosis biomarkers**			
GO:0005515	Protein binding	131	4.67 × 10^−29^
GO:0005102	Receptor binding	45	1.18 × 10^−11^
GO:0044877	Macromolecular complex binding	44	1.98 × 10^−11^
GO:0005488	Binding	160	2.91 × 10^−9^
GO:0042802	Identical protein binding	35	1.63 × 10^−7^
GO:0019899	Enzyme binding	41	5.39 × 10^−7^
GO:0032403	Protein complex binding	25	6.42 × 10^−7^
GO:0003684	Damaged DNA binding	9	8.98 × 10^−6^
GO:0043566	Structure-specific DNA binding	15	1.09 × 10^−5^
GO:0019900	Kinase binding	19	9.05 × 10^−5^

**Table 4:** GO analysis results in cellular component level for CRC protein biomarkers.

Pathway ID	Pathway Description	Counts	FDR
**A. GO analysis in cellular component level for diagnosis biomarkers**			
GO:0005615	Extracellular space	20	1.49 × 10^−6^
GO:0022627	Cytosolic small ribosomal subunit	6	1.49 × 10^−6^
GO:0031982	Vesicle	33	1.49 × 10^−6^
GO:0031988	Membrane-bounded vesicle	32	2.34 × 10^−6^
GO:0005576	Extracellular region	36	2.74 × 10^−6^
GO:0044421	Extracellular region part	32	7.96 × 10^−6^
GO:0034774	Secretory granule lumen	6	1.67 × 10^−5^
GO:0022626	Cytosolic ribosome	6	8.62 × 10^−5^
GO:0030141	Secretory granule	9	8.62 × 10^−5^
GO:0031093	Platelet alpha granule lumen	5	8.62 × 10^−5^
**B. GO analysis in cellular component level for treatment biomarkers**			
GO:0005829	Cytosol	24	4.63 × 10^−5^
GO:0044428	Nuclear part	26	4.63 × 10^−5^
GO:0032991	Macromolecular complex	27	0.000117
GO:0043233	Organelle lumen	26	0.000117
GO:0043234	Protein complex	25	0.000117
GO:0044427	Chromosomal part	11	0.000117
GO:0031981	Nuclear lumen	23	0.000149
GO:0005654	Nucleoplasm	21	0.000153
GO:0005694	Chromosome	11	0.000164
GO:0070013	Intracellular organelle lumen	24	0.000662
**C. GO analysis in cellular component level for prognosis biomarkers**			
GO:0005576	Extracellular region	96	1.33 × 10^−10^
GO:0005615	Extracellular space	46	1.62 × 10^−10^
GO:0044421	Extracellular region part	85	1.96 × 10^−10^
GO:0005829	Cytosol	72	6.05 × 10^−8^
GO:0005912	Adherens junction	23	1.13 × 10^−7^
GO:0005924	Cell-substrate adherens junction	21	2.33 × 10^−7^
GO:0043227	Membrane-bounded organelle	163	3.07 × 10^−7^
GO:0009986	Cell surface	28	3.94 × 10^−7^
GO:0005925	Focal adhesion	20	6.84 × 10^−7^
GO:0031982	Vesicle	73	1.04 × 10^−6^

**Table 5:** Biological functional analysis for overlapping DNA transferred by prognosis biomarkers.

Pathway ID	Pathway Description	Counts	FDR
**A. KEGG pathway enrichment for overlapping DNA transferred by prognosis biomarkers**			
05206	MicroRNAs in cancer	5	0.000171
04068	FoxO signalling pathway	3	0.0466
05200	Pathways in cancer	4	0.0466
**B. GO analysis result in biological process level for overlapping DNA transferred by prognosis biomarkers**			
GO:0009887	Organ morphogenesis	9	0.00107
GO:0010468	Regulation of gene expression	16	0.00107
GO:0010557	Positive regulation of macromolecule biosynthetic process	11	0.00107
GO:0010628	Positive regulation of gene expression	11	0.00107
GO:2000112	Regulation of cellular macromolecule biosynthetic process	15	0.00107
GO:0031328	Positive regulation of cellular biosynthetic process	11	0.00118
GO:0048514	Blood vessel morphogenesis	6	0.00514
GO:0010556	Regulation of macromolecule biosynthetic process	14	0.00588
GO:0010604	Positive regulation of macromolecule metabolic process	12	0.00608
GO:0001568	Blood vessel development	6	0.00631

## References

[1] Siegel R.L., Miller K.D., Jemal A. (2018). Cancer statistics, 2018. CA Cancer J. Clin..

[2] Siegel R.L., Miller K.D., Fedewa S.A., Ahnen D.J., Meester R.G.S., Barzi A. (2017). Colorectal cancer statistics, 2017. CA Cancer J. Clin..

[3] Shah R., Jones E., Vidart V., Kuppen P.J., Conti J.A., Francis N.K. (2014). Biomarkers for early detection of colorectal cancer and polyps: Systematic review. Cancer Epidemiol. Biomark. Prev..

[4] SEER. http://seer.cancer.gov/statfacts/html/colorect.html.

[5] Brenner H., Kloor M., Pox C.P. (2014). Colorectal cancer. Lancet.

[6] Center M.M., Jemal A., Smith R.A., Ward E. (2009). Worldwide variations in colorectal cancer. CA Cancer J. Clin..

[7] Shin S.H., Bode A.M., Dong Z. (2017). Precision medicine: The foundation of future cancer therapeutics. NPJ Precis. Oncol..

[8] Henry N.L., Hayes D.F. (2012). Cancer biomarkers. Mol. Oncol..

[9] Zhang X., Sun X.F., Cao Y., Ye B., Peng Q., Liu X., Shen B., Zhang H. (2018). CBD: A biomarker database for colorectal cancer. Database.

[10] Schirripa M., Lenz H.J. (2016). Biomarker in Colorectal Cancer. Cancer J..

[11] Lin Y., Qian F., Shen L., Chen F., Chen J., Shen B. (2017). Computer-aided biomarker discovery for precision medicine: Data resources, models and applications. Brief. Bioinform..

[12] Lobdell D.T., Mendola P. (2005). Development of a biomarkers database for the National Children’s Study. Toxicol. Appl. Pharmacol..

[13] Yerlikaya S., Broger T., MacLean E., Pai M., Denkinger C.M. (2017). A tuberculosis biomarker database: The key to novel TB diagnostics. Int. J. Infect. Dis..

[14] Yang I.S., Ryu C., Cho K.J., Kim J.K., Ong S.H., Mitchell W.P., Kim B.S., Oh H.B., Kim K.H. (2008). IDBD: Infectious disease biomarker database. Nucleic Acids Res..

[15] Dai H.J., Wu J.C., Lin W.S., Reyes A.J., Dela Rosa M.A., Syed-Abdul S., Tsai R.T., Hsu W.L. (2014). LiverCancerMarkerRIF: A liver cancer biomarker interactive curation system combining text mining and expert annotations. Database.

[16] Osumi H., Shinozaki E., Suenaga M., Matsusaka S., Konishi T., Akiyoshi T., Fujimoto Y., Nagayama S., Fukunaga Y., Ueno M. (2016). RAS mutation is a prognostic biomarker in colorectal cancer patients with metastasectomy. Int. J. Cancer.

[17] Sun X.F., Carstensen J.M., Zhang H., Stal O., Wingren S., Hatschek T., Nordenskjold B. (1992). Prognostic significance of cytoplasmic p53 oncoprotein in colorectal adenocarcinoma. Lancet.

[18] Wang M.J., Ping J., Li Y., Adell G., Arbman G., Nodin B., Meng W.J., Zhang H., Yu Y.Y., Wang C. (2015). The prognostic factors and multiple biomarkers in young patients with colorectal cancer. Sci Rep..

[19] Pathak S., Meng W.J., Nandy S.K., Ping J., Bisgin A., Helmfors L., Waldmann P., Sun X.F. (2015). Radiation and SN38 treatments modulate the expression of microRNAs, cytokines and chemokines in colon cancer cells in a p53-directed manner. Oncotarget.

[20] Sun X.F., Ekberg H., Zhang H., Carstensen J.M., Nordenskjold B. (1998). Overexpression of ras is an independent prognostic factor in colorectal adenocarcinoma. APMIS.

[21] Sun X.F., Bartik Z., Zhang H. (2003). Bcl-2 expression is a prognostic factor in the subgroups of patients with colorectal cancer. Int. J. Oncol..

[22] Iseki Y., Shibutani M., Maeda K., Nagahara H., Ikeya T., Hirakawa K. (2017). Significance of E-cadherin and CD44 expression in patients with unresectable metastatic colorectal cancer. Oncol. Lett..

[23] Ning S., Wei W., Li J., Hou B., Zhong J., Xie Y., Liu H., Mo X., Chen J., Zhang L. (2018). Clinical significance and diagnostic capacity of serum TK1, CEA, CA 19-9 and CA 72-4 levels in gastric and colorectal cancer patients. J. Cancer.

[24] Yuan X., Chen J., Lin Y., Li Y., Xu L., Chen L., Hua H., Shen B. (2017). Network Biomarkers Constructed from Gene Expression and Protein-Protein Interaction Data for Accurate Prediction of Leukemia. J. Cancer.

[25] McGuire A., Brown J.A., Kerin M.J. (2015). Metastatic breast cancer: The potential of miRNA for diagnosis and treatment monitoring. Cancer Metastasis. Rev..

[26] Shin V.Y., Chu K.M. (2014). MiRNA as potential biomarkers and therapeutic targets for gastric cancer. World J. Gastroenterol..

[27] De Robertis M., Poeta M.L., Signori E., Fazio V.M. (2018). Current understanding and clinical utility of miRNAs regulation of colon cancer stem cells. Semin. Cancer Biol..

[28] Xiao J., Jing Z.C., Ellinor P.T., Liang D., Zhang H., Liu Y., Chen X., Pan L., Lyon R., Liu Y. (2011). MicroRNA-134 as a potential plasma biomarker for the diagnosis of acute pulmonary embolism. J. Transl. Med..

[29] Rong H., Liu T.B., Yang K.J., Yang H.C., Wu D.H., Liao C.P., Hong F., Yang H.Z., Wan F., Ye X.Y. (2011). MicroRNA-134 plasma levels before and after treatment for bipolar mania. J. Psychiatr. Res..

[30] Wang X., Luo Y., Liu S., Tan L., Wang S., Man R. (2017). MicroRNA-134 plasma levels before and after treatment with valproic acid for epilepsy patients. Oncotarget.

[31] O’Brien K., Lowry M.C., Corcoran C., Martinez V.G., Daly M., Rani S., Gallagher W.M., Radomski M.W., MacLeod R.A., O’Driscoll L. (2015). miR-134 in extracellular vesicles reduces triple-negative breast cancer aggression and increases drug sensitivity. Oncotarget.

[32] Wang T., Lv M., Shen S., Zhou S., Wang P., Chen Y., Liu B., Yu L., Hou Y. (2012). Cell-free microRNA expression profiles in malignant effusion associated with patient survival in non-small cell lung cancer. PLoS ONE.

[33] Lu G., Sun Y., An S., Xin S., Ren X., Zhang D., Wu P., Liao W., Ding Y., Liang L. (2015). MicroRNA-34a targets FMNL2 and E2F5 and suppresses the progression of colorectal cancer. Exp. Mol. Pathol..

[34] Zhu M., Huang Z., Zhu D., Zhou X., Shan X., Qi L.W., Wu L., Cheng W., Zhu J., Zhang L. (2017). A panel of microRNA signature in serum for colorectal cancer diagnosis. Oncotarget.

[35] Carpelan-Holmstrom M.A., Haglund C.H., Roberts P.J. (1996). Differences in serum tumor markers between colon and rectal cancer. Comparison of CA 242 and carcinoembryonic antigen. Dis. Colon Rectum..

[36] Han M., Liew C.T., Zhang H.W., Chao S., Zheng R., Yip K.T., Song Z.Y., Li H.M., Geng X.P., Zhu L.X. (2008). Novel blood-based, five-gene biomarker set for the detection of colorectal cancer. Clin. Cancer Res..

[37] Komuro Y., Watanabe T., Tsurita G., Muto T., Nagawa H. (2005). Evaluating the combination of molecular prognostic factors in tumor radiosensitivity in rectal cancer. Hepatogastroenterology.

[38] Nakajima T.E., Yamada Y., Shimoda T., Matsubara J., Kato K., Hamaguchi T., Shimada Y., Okayama Y., Oka T., Shirao K. (2008). Combination of O6-methylguanine-DNA methyltransferase and thymidylate synthase for the prediction of fluoropyrimidine efficacy. Eur. J. Cancer.

[39] Chen H., Sun X., Ge W., Qian Y., Bai R., Zheng S. (2017). A seven-gene signature predicts overall survival of patients with colorectal cancer. Oncotarget.

[40] Ge J., Chen Z., Li R., Lu T., Xiao G. (2014). Upregulation of microRNA-196a and microRNA-196b cooperatively correlate with aggressive progression and unfavorable prognosis in patients with colorectal cancer. Cancer Cell Int..

[41] Tatsuta S., Tanaka S., Haruma K., Yoshihara M., Sumii K., Kajiyama G., Shimamoto F. (1997). Combined expression of urokinase-type plasminogen activator and proliferating cell nuclear antigen at the deepest invasive portion correlates with colorectal cancer prognosis. Int. J. Oncol..

[42] Hu Y., Gaedcke J., Emons G., Beissbarth T., Grade M., Jo P., Yeager M., Chanock S.J., Wolff H., Camps J. (2018). Colorectal cancer susceptibility loci as predictive markers of rectal cancer prognosis after surgery. Genes Chromosomes Cancer.

[43] Wang X. (2016). Improving microRNA target prediction by modeling with unambiguously identified microRNA-target pairs from CLIP-ligation studies. Bioinformatics.

